# Occurrence Proportion and Associated Factors of Respiratory Failure During Hospitalization in Chinese Patients With AECOPD: A Systematic Review and Meta‐Analysis

**DOI:** 10.1111/crj.70237

**Published:** 2026-10-22

**Authors:** Dandan Liu, Minzuo Chen, Kaosi Wen, Jingmei Pan, Xiangyang Duan, Tao Yang, Shengru Tao

**Affiliations:** ^1^ School of Nursing Jinan University Guangzhou Guangdong China; ^2^ The First Affiliated Hospital of Jinan University Guangzhou Guangdong China

**Keywords:** acute exacerbation of chronic obstructive pulmonary disease, associated factors, meta‐analysis, occurrence proportion, respiratory failure

## Abstract

**Objective:**

To systematically evaluate the occurrence proportion and associated factors of respiratory failure during hospitalization in Chinese patients with acute exacerbation of chronic obstructive pulmonary disease (AECOPD).

**Methods:**

Eight databases (CNKI, Wanfang, VIP, CBM, PubMed, Web of Science, Embase, and Cochrane Library) were searched from inception to March 1, 2026. Studies reporting multivariable‐adjusted odds ratios (ORs) for factors associated with respiratory failure in AECOPD patients were included. The Newcastle–Ottawa Scale was used for quality assessment. A single‐group rate meta‐analysis with Freeman–Tukey double arcsine transformation was performed to pool the occurrence proportion. Subgroup analyses were conducted by study design, geographical region (Qinling–Huaihe Line), and respiratory failure type. Factors associated with respiratory failure were stratified by definitions and cutoff values; only subgroups with ≥ 3 studies were pooled.

**Results:**

Twenty‐three studies (5 cohort, 18 case–control) with 4212 patients were included. The pooled occurrence proportion of respiratory failure was 41% (95% CI: 0.37–0.46). Meta‐analysis identified nine factors associated with respiratory failure: comorbid cardiac disease (OR = 2.12, 95% CI: 1.68–2.67), advanced age (OR = 1.22, 95% CI: 1.13–1.33), elevated NLR (OR = 1.87, 95% CI: 1.50–2.33), elevated PCT (OR = 2.27, 95% CI: 1.29–4.00), elevated D‐dimer (OR = 1.72, 95% CI: 1.01–2.90), ≥ 2 annual exacerbations (OR = 2.48, 95% CI: 1.71–3.60), higher APACHE II score (OR = 1.29, 95% CI: 1.17–1.43), elevated CRP (OR = 1.13, 95% CI: 1.02–1.24), and decreased FEV1% predicted (OR = 0.81, 95% CI: 0.67–0.99). Model dependence analysis showed that ALB was statistically significant only under the fixed‐effects model but not under the random‐effects model. NE was not statistically significant under either model. Therefore, ALB and NE were not included among the nine factors that were statistically significant in the primary analysis.

**Conclusion:**

The occurrence proportion of respiratory failure in hospitalized Chinese AECOPD patients is high and associated with comorbidities, clinical characteristics, severity scores, and inflammatory/coagulation markers. These findings support risk identification and monitoring, but prospective studies are needed to validate their predictive and interventional value.

## Graphical abstract

This meta‐analysis of 23 studies with 4212 Chinese AECOPD patients found a 41% occurrence proportion of respiratory failure, with substantial heterogeneity. Nine associated factors were identified, including cardiac disease, advanced age, elevated biomarkers, frequent exacerbations, higher APACHE II score, and decreased FEV_1_%pred. These findings support risk stratification and targeted monitoring, but causality cannot be inferred and the estimate should not be interpreted as population incidence.

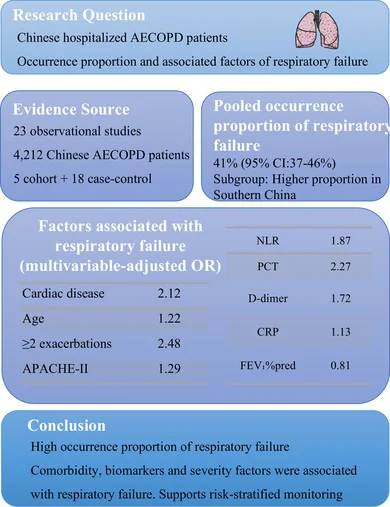

## 1 Introduction

Chronic obstructive pulmonary disease (COPD) is a highly prevalent chronic respiratory disease worldwide characterized by persistent airflow limitation; it has a protracted course and poor prognosis and was ranked as the third leading cause of death globally by the Global Burden of Disease Study 2019 [1, 2, 3]. Acute exacerbation of chronic obstructive pulmonary disease (AECOPD) is the most important acute adverse event during the course of COPD, characterized by acute worsening of respiratory symptoms such as cough, sputum production, and dyspnea, which not only leads to progressive decline in lung function but also significantly increases hospitalization rates, readmission rates, and healthcare resource consumption [4, 5].

AECOPD is frequently complicated by multiple comorbidities, including respiratory failure, respiratory tract infection, heart failure, pulmonary embolism, and anemia [6]. Among these, respiratory failure is one of the most common and most critical complications of AECOPD; patients may rapidly develop severe hypoxemia and carbon dioxide retention, often requiring mechanical ventilation support, which prolongs hospital stay, increases medical costs, and significantly worsens short‐ and long‐term prognosis [7, 8]. AECOPD complicated by respiratory failure significantly increases the tracheal intubation rate and mortality, and in severe cases may lead to multiple organ dysfunction [9, 10]. Therefore, respiratory failure is not only a key pathophysiological link in the progression of AECOPD but also a direct driver of poor prognosis.

Early identification of factors associated with respiratory failure in patients with AECOPD and the implementation of targeted measures are of great significance for improving patient prognosis. Previous systematic reviews have synthesized factors associated with respiratory failure in AECOPD, but they still have the following limitations: most systematic reviews included studies conducted worldwide and did not separately pool data for Chinese patients with AECOPD, despite differences in the etiology spectrum, diagnosis and treatment pathways, and comorbidity spectrum between Chinese COPD patients and Western populations [11, 12]; most previous studies did not strictly distinguish between univariate analysis and multivariable‐adjusted effect estimates when pooling factors associated with respiratory failure; univariate analysis does not control for confounding factors and may lead to bias in effect estimation [13, 14]; in addition, China has a vast territory, and there are certain differences in climate and pathogen spectrum between the northern and southern regions [15]; however, previous studies have not performed geographical region‐stratified analysis of the occurrence proportion of respiratory failure in AECOPD.

Therefore, referring to the guidelines for meta‐analysis of observational studies [16], this study estimated the pooled occurrence proportion based on cohort studies and case–control studies, conducted strict subgroup stratification according to indicator definitions and cutoff values, included only multivariable‐adjusted ORs in the meta‐analysis of factors associated with respiratory failure, and performed subgroup analyses by type of respiratory failure and geographical region, so as to systematically evaluate factors associated with respiratory failure in Chinese patients with AECOPD, with the aim of providing reliable evidence‐based support for clinical risk assessment, early warning, and individualized intervention.

## 2 Materials and Methods

This systematic review and meta‐analysis was conducted in accordance with the PRISMA 2020 guidelines and reported according to the PRISMA 2020 checklist [17]. The study protocol was registered on the PROSPERO platform (registration number: CRD420261405751).

### 2.1 Inclusion and Exclusion Criteria

The inclusion criteria were as follows: (1) Study population: hospitalized patients with a confirmed diagnosis of AECOPD in China, meeting the diagnostic criteria for AECOPD [18], who were divided into a case group and a control group according to whether respiratory failure was present; (2) study design: case–control studies or cohort studies; case–control studies were not used to estimate population incidence. They were included only to describe the occurrence proportion of respiratory failure among hospitalized AECOPD patients and to explore associated factors; (3) outcome: whether respiratory failure newly occurred after admission or during hospitalization, including type I respiratory failure, type II respiratory failure, and mixed respiratory failure. The diagnostic criteria [19] were defined as: at sea level and at rest, arterial partial pressure of oxygen (PaO_2_) < 60 mmHg with normal or decreased arterial partial pressure of carbon dioxide (PaCO_2_); or PaO_2_ < 60 mmHg with PaCO_2_ > 50 mmHg; or both; (4) exposure factors: factors associated with respiratory failure in patients with AECOPD; (5) data requirements: studies had to provide multivariable logistic or Cox regression‐adjusted odds ratios (ORs) with 95% confidence intervals (CIs), and each study had to allow extraction of the number of respiratory failure cases and the total number of patients. The exclusion criteria were as follows: (1) reviews, systematic reviews, meta‐analyses, case reports, conference abstracts, animal experiments, and dissertations/theses; (2) studies that only provided univariate analysis results without multivariable adjustment; (3) studies with missing data, duplicate publication, inability to extract effect sizes, or no effect size pooling.

### 2.2 Literature Search Strategy

The search strategy combined subject headings and free‐text terms, using keywords including “chronic obstructive pulmonary disease”, “COPD exacerbation”, “acute exacerbation of COPD”, “AECOPD”, “respiratory failure”, “type 1 respiratory failure”, “type 2 respiratory failure”, “hypercapnic respiratory failure”, “hypoxemic respiratory failure”, “acute‐on‐chronic respiratory failure”, “COPD‐related respiratory failure”, “risk factors”, “risk factor”, “influencing factors”, “incidence”, and “prevalence” to search the databases of CNKI, CBM, VIP, Wanfang, PubMed, Embase, Web of Science, and the Cochrane Library. The search period was from the inception of each database to March 1, 2026. The search strategy for PubMed is presented as an example in Table 1.

**TABLE 1:** PubMed database search strategy. The search strategy combined MeSH terms and free‐text terms using Boolean operators. The search period was from database inception to March 1, 2026.

No	Search strategy
#1	“Pulmonary Disease, Chronic Obstructive”[MeSH Terms] OR (“COPD exacerbation” [Title/Abstract] OR “acute exacerbation of COPD” [Title/Abstract] OR AECOPD [Title/Abstract])
#2	“Respiratory Insufficiency”[MeSH Terms] OR (“respiratory failure”[Title/Abstract] OR “type 1 respiratory failure”[Title/Abstract] OR “type 2 respiratory failure”[Title/Abstract] OR “hypercapnic respiratory failure”[Title/Abstract] OR “hypoxemic respiratory failure”[Title/Abstract])
#3	“risk factors”[Title/Abstract] OR “risk factor”[Title/Abstract] OR “influencing factors”[Title/Abstract] OR “Occurrence proportion” [Title/Abstract] OR “Prevalence” [Title/Abstract]
#4	#1 AND #2 AND #3

### 2.3 Literature Screening and Data Extraction

In this study, NoteExpress was used for literature management; two researchers independently screened the literature according to the inclusion and exclusion criteria, and any disagreements were resolved by a third researcher. After eligible studies were identified, data were systematically extracted from the screened literature using a standardized electronic spreadsheet to ensure comprehensive and standardized information collection. The extracted data included first author, publication year, study country, study design, sample size (case group vs. control group), and factors associated with respiratory failure in patients with AECOPD, specifically including comorbid cardiac disease, forced expiratory volume in 1‐s percent predicted (FEV_1_%pred) on admission, age, number of AECOPD exacerbations per year, serum albumin level (ALB), C‐reactive protein (CRP), procalcitonin (PCT), neutrophil‐to‐lymphocyte ratio (NLR), D‐dimer, neutrophil percentage (NE), and Acute Physiology and Chronic Health Evaluation II (APACHE II) score (Table 2).

**TABLE 2:** Subgroup analysis of the occurrence proportion of respiratory failure in patients with AECOPD. Data are presented as pooled occurrence proportion with 95% confidence intervals. Q_b represents the test statistic for between‐subgroup differences. A random‐effects model with Freeman–Tukey double arcsine transformation was used for all analyses.

Subgroup	No. of studies	Pooled occurrence proportion	95% CI	Heterogeneity		Test for subgroup differences
				*I* ^2^ (%)	*p*	
Study design						
Case–control	18	0.40	0.36–0.45	85.4	< 0.001	
Cohort	5	0.45	0.32–0.58	96.3	< 0.001	
Between‐group comparison						Q_b = 0.41, df = 1, *p* = 0.523
Geographic region*						
North	5	0.37	0.26–0.47	89.4	< 0.001	
Central	7	0.35	0.31–0.39	49.2	0.07	
South	11	0.48	0.41–0.54	90.9	< 0.001	
Between‐group comparison						Q_b = 10.28, df = 2, *p* = 0.006
Respiratory failure type						
Type I respiratory failure	2	0.45	0.39–0.51	0.0	0.393	
Type II respiratory failure	8	0.38	0.30–0.47	92.4	< 0.001	
Mixed respiratory failure	13	0.43	0.36–0.49	90.8	< 0.001	
Between‐group comparison						Q_b = 1.63, df = 2, *p* = 0.442

* Geographic regions were divided according to the Qinling–Huaihe Line: North (Hebei, Shandong, Liaoning, Henan), Central (Anhui), South (Jiangsu, Sichuan, Guangdong, Hainan).

Definitions and diagnostic criteria of each associated factor: The factors associated with respiratory failure extracted in this study were based on the definitions in each original study, without setting additional unified cutoff values. Among them, (1) age was a continuous variable; in all studies, the actual age (years) of patients was included in multivariable regression models as a continuous independent variable, and the effect estimate (OR) reflected the change in the risk of respiratory failure associated with each 1‐year increase in age; (2) comorbid cardiac disease was defined as clearly diagnosed coronary heart disease, chronic heart failure, arrhythmia, pulmonary heart disease, or other cardiovascular comorbidities present before or at admission; it was extracted based on medical history records in the electronic medical record system, with the specific diagnostic criteria of each original study being followed; (3) the cutoff values and detection methods for the remaining factors associated with respiratory failure were extracted according to the definitions in each original study.

### 2.4 Literature Quality Assessment

The Newcastle–Ottawa Scale (NOS) was used to assess the quality of observational studies, with scoring across three domains—selection of study subjects, comparability between groups, and outcome assessment—comprising eight items, with a total score ranging from 0 to 9; studies with scores ≤ 4 were considered low quality, 5–6 moderate quality and ≥ 7 high quality [20].

### 2.5 Statistical Analysis

Meta‐analysis was performed using Stata 18.0 statistical software. The Freeman–Tukey double arcsine transformation was used to perform a single‐group rate meta‐analysis, and a fixed‐effects or random‐effects model was selected according to the heterogeneity across studies to pool the occurrence proportion. Because case–control studies select participants by outcome status, they cannot provide valid estimates of population incidence. To examine the influence of study design on the pooled occurrence proportion, subgroup analysis by study design (cohort vs. case–control) was performed. This approach retains all eligible studies and directly compares estimates across study designs. The pooled proportion is therefore interpreted as a descriptive occurrence proportion among hospitalized AECOPD patients, not as incidence. For the analysis of factors associated with respiratory failure, multivariable‐adjusted ORs and 95% CIs from each study were used as the effect estimates; the Q test combined with the *I* ^2^ statistic was used to assess between‐study heterogeneity: when *I* ^2^ ≤ 50% and *p* > 0.10, heterogeneity was considered low and a fixed‐effects model was selected; when *I* ^2^ > 50% or *p* ≤ 0.10, significant heterogeneity was considered present and a random‐effects model was used.

The meta‐analysis of factors associated with respiratory failure was stratified strictly according to different definitions and cutoff values of the factors; only subgroups with three or more included studies were pooled and subgroups with fewer than three studies were excluded.

To analyze the impact of different study design types on the occurrence proportion of respiratory failure in patients with AECOPD, this study conducted a subgroup analysis by study type, dividing the included studies into two subgroups: cohort studies and case–control studies. Second, to analyze the impact of geographical region on the occurrence proportion, this study conducted subgroup analysis by climatic‐geographical region. According to the climatic‐geographical division of China (the Qinling–Huaihe Line), the included studies were divided into three subgroups: northern, central, and southern; the northern subgroup included Hebei, Shandong, Liaoning, and Henan; the central subgroup was Anhui; and the southern subgroup included Jiangsu, Nanjing, Sichuan, Guangdong, and Hainan [21]. In addition, considering differences in the diagnostic criteria for respiratory failure across original studies, this study further performed subgroup analysis by type of respiratory failure, following the diagnostic criteria proposed by the Global Initiative for Chronic Obstructive Lung Disease (GOLD) and the Chinese Thoracic Society [4, 6], and divided the included studies into the following three subgroups: (1) type I respiratory failure subgroup: arterial blood gas analysis showing PaO_2_ < 60 mmHg with normal or decreased PaCO_2_; (2) type II respiratory failure subgroup: arterial blood gas analysis simultaneously showing PaO_2_ < 60 mmHg and PaCO_2_ > 50 mmHg; (3) mixed respiratory failure subgroup: arterial blood gas analysis primarily based on PaO_2_ < 60 mmHg, but without clear distinction of whether PaCO_2_ was elevated, or the original description was “with or without” elevated PaCO_2_. All the above analyses used random‐effects models to pool the occurrence proportion of respiratory failure in each subgroup, and the Q‐test for subgroup differences was used to compare between‐subgroup differences. For a specific subgroup, if the number of included studies was less than three, no statistical pooling was performed for that subgroup, and its results were only descriptively reported to avoid estimation bias due to insufficient statistical power.

To assess the dependence of pooled results on the choice of effect model, this study conducted model dependence analysis between fixed‐effects and random‐effects models. For factors associated with respiratory failure with ≥ 7 included studies, funnel plots combined with Begg's rank correlation test were used to explore small‐study effects; for the occurrence proportion of respiratory failure, funnel plots combined with Egger's regression test were used. The statistical significance level was set at *α* = 0.05 for all tests.

## 3 Results

### 3.1 Literature Screening Process and Results

The initial search identified a total of 3191 records, of which 614 duplicates were removed, leaving 2577 records; after screening titles and abstracts, 2383 ineligible records were excluded, leaving 194 records; after full‐text review, studies with incomplete data, lack of multivariable adjustment, duplicate publication, and other reasons were excluded, and 23 studies were ultimately included. The literature screening process is shown in Figure 1.

**FIGURE 1:**
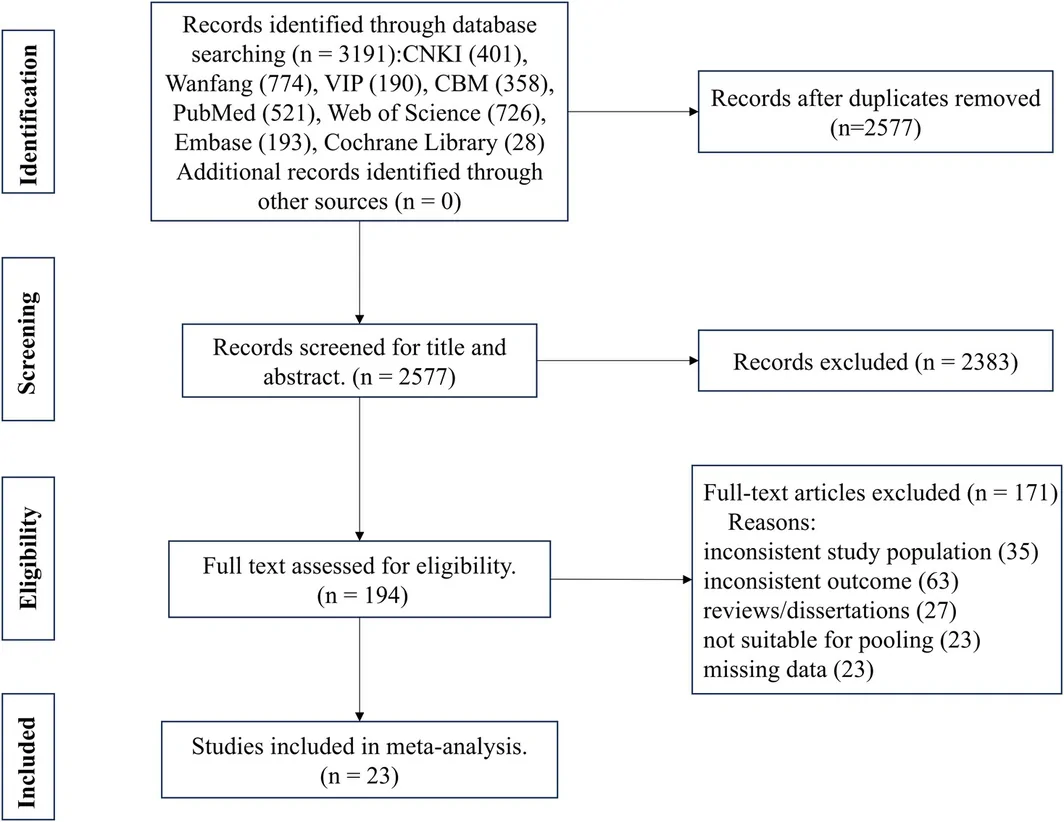
PRISMA 2020 flow diagram of the literature screening process. Each box represents a step in the screening process, with the number of records remaining at each stage indicated. Arrows connect sequential steps, showing the flow from initial identification through duplicate removal, title/abstract screening, full‐text assessment, and final inclusion. The reasons for exclusion at the full‐text stage are also presented. The diagram illustrates that after sequential screening, 23 studies were ultimately included in the meta‐analysis.

### 3.2 Basic Characteristics of Included Studies

The 23 included studies were all conducted in Chinese populations, with publication years ranging from 2018 to 2025; there were 5 retrospective cohort studies [22, 23, 24, 25, 26] and 18 case–control studies [27, 28, 29, 30, 31, 32, 33, 34, 35, 36, 37, 38, 39, 40, 41, 42, 43, 44]. The total sample size was 4212 patients, including 2486 patients with AECOPD alone and 1726 patients with AECOPD and respiratory failure. A total of 11 factors associated with respiratory failure in patients with AECOPD were identified. The results of literature quality assessment showed that 14 studies [22, 23, 25, 26, 27, 31, 34, 36, 37, 39, 40, 41, 43, 44] had NOS scores of 5–6, and 9 studies [24, 28, 29, 30, 32, 33, 35, 38, 42] had NOS scores ≥ 7, indicating overall high quality. The basic characteristics and NOS scores of the included studies are shown in Table S1.

### 3.3 Meta‐Analysis Results of the Occurrence Proportion of Respiratory Failure

This study performed a single‐group rate meta‐analysis of the occurrence proportion of respiratory failure based on the included 5 cohort studies and 18 case–control studies. The heterogeneity test showed high heterogeneity among studies (*I* ^2^ = 90.4%, *p* < 0.001); therefore, a random‐effects model was used for pooling. The meta‐analysis showed (Figure 2) that the pooled occurrence proportion of respiratory failure in patients with AECOPD was 41% (95% CI: 0.37–0.46, *p* < 0.05). Subgroup analysis by study design showed no significant difference between cohort and case–control studies (Q_b = 0.41, df = 1, *p* = 0.523), supporting the robustness of the descriptive pooled occurrence proportion.

**FIGURE 2:**
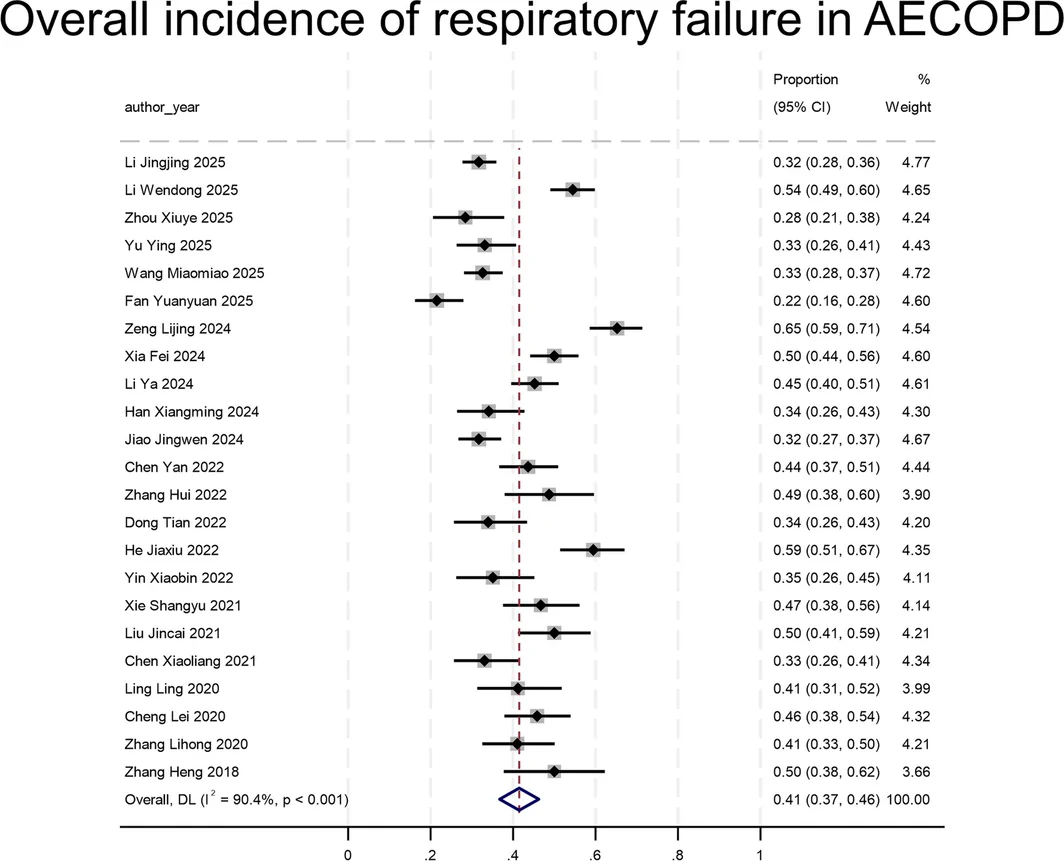
Forest plot of the pooled occurrence proportion of respiratory failure in patients with AECOPD. Each square represents the occurrence proportion of an individual study, with the size of the square proportional to the weight assigned in the random‐effects model. Horizontal lines indicate 95% confidence intervals. The diamond at the bottom represents the pooled occurrence proportion estimate, with its width indicating the 95% confidence interval. The *I* ^2^ statistic and the *p*‐value for heterogeneity are displayed, providing a measure of between‐study variability. The plot summarizes the occurrence proportion estimates from all included studies and provides the overall pooled estimate, showing considerable variation across studies.

### 3.4 Subgroup Analysis Results

Stratified by study design type, the pooled occurrence proportion was 40% (95% CI: 0.36–0.45) among 18 case–control studies and 45% (95% CI: 0.32–0.58) among 5 cohort studies (Table 2). The between‐subgroup difference was not statistically significant (Q_b = 0.41, df = 1, *p* = 0.523) (Figure S1).

Stratified by geographical region, the pooled occurrence proportion was 37% (95% CI: 0.26–0.47) in the northern region (5 studies), 35% (95% CI: 0.31–0.39) in the central region (7 studies), and 48% (95% CI: 0.41–0.54) in the southern region (11 studies). Study‐level subgroup comparison suggested a higher occurrence proportion in the southern region (Q_b = 10.28, df = 2, *p* = 0.006). This difference should be interpreted as a study‐level association and may be influenced by single‐center sources, study years, patient composition, sampling methods, and diagnostic/treatment practices. It cannot be attributed to climatic or pathogen differences (Figure S2).

Stratified by the diagnostic criteria for respiratory failure, the pooled occurrence proportion was 45% (95% CI: 0.39–0.51) in 2 studies with strict definition of type I respiratory failure, 38% (95% CI: 0.30–0.47) in 8 studies with strict definition of type II respiratory failure, and 43% (95% CI: 0.36–0.49) in 13 studies using a mixed definition. The between‐subgroup difference was not statistically significant (Q_b = 1.63, df = 2, *p* = 0.442) (Figure S3).

### 3.5 Meta‐Analysis Results of Factors Associated With Respiratory Failure

A total of 11 factors associated with respiratory failure were included in the pooled analysis, and comorbid cardiac disease, advanced age, elevated NLR, elevated PCT, elevated D‐dimer, annual exacerbation frequency ≥ 2, higher APACHE II score, elevated CRP, and decreased FEV_1_%pred were identified as associated factors for respiratory failure in patients with AECOPD (Table 3) (Figures S4–S14). ALB was statistically significant only under the fixed‐effects model but not under the random‐effects model. NE was not statistically significant under either model. Therefore, ALB and NE were not included among the nine factors that were statistically significant in the primary analysis.

**TABLE 3:** Meta‐analysis of factors associated with respiratory failure secondary to AECOPD. OR, odds ratio; CI, confidence interval; FEV_1_%pred, forced expiratory volume in 1‐s percent predicted; NLR, neutrophil‐to‐lymphocyte ratio; PCT, procalcitonin; CRP, C‐reactive protein; ALB, serum albumin; NE, neutrophil percentage. An OR > 1 indicates higher odds of respiratory failure, whereas an OR < 1 indicates lower odds of respiratory failure. Fixed‐effects or random‐effects models were selected based on heterogeneity test results.

Associated factor	No. of studies	Heterogeneity test		Effect model	Pooled effect size			
		*p*	*I* ^2^%		OR	95% CI		*p*
Combined cardiac disease	8	0.12	38.58	Fixed	2.12	1.68	2.67	0.00
FEV_1_%pred	4	0.02	89.24	Random	0.81	0.67	0.99	0.04
Age	3	0.41	0.00	Fixed	1.22	1.13	1.33	0.00
NLR	3	0.32	11.81	Fixed	1.87	1.50	2.33	0.00
PCT	3	0.02	73.33	Random	2.27	1.29	4.00	0.00
D‐dimer	3	0.02	89.61	Random	1.72	1.01	2.90	0.00
Annual exacerbation frequency ≥ 2	3	0.46	0.00	Fixed	2.48	1.71	3.60	0.00
APACHE II score	3	0.20	38.52	Fixed	1.29	1.17	1.43	0.00
CRP	3	0.07	63.50	Random	1.13	1.02	1.24	0.01
ALB	3	0.00	95.86	Random	0.21	0.03	1.43	0.11
NE	3	0.00	94.13	Random	2.07	0.90	4.79	0.09

### 3.6 Sensitivity Analysis

Model dependence analysis showed that ALB was statistically significant under the fixed‐effects model but not under the random‐effects model. NE was not statistically significant under either model. Therefore, ALB and NE were not included among the nine factors that were statistically significant in the primary analysis. For the remaining factors, effect direction and statistical significance were generally stable across models (Table S2).

### 3.7 Publication Bias

Funnel plot combined with Egger's regression test was used to explore small‐study effects for the occurrence proportion of respiratory failure during hospitalization in patients with AECOPD (Figure 3). The intercept of Egger's regression test was 1.468 (standard error 2.460, *t* = 0.60, *p* = 0.557), indicating no statistically significant small‐study effects. However, there was high heterogeneity among the included studies, and interpretation of the funnel plot and Egger's test should still be cautious. For comorbid cardiac disease as a factor associated with respiratory failure, funnel plot combined with Begg's rank correlation test was used for exploration (Figure 4). The results of Begg's test showed Kendall's score = 20.00, *z* = 2.35, and *p* = 0.0187, suggesting the possible presence of small‐study effects or publication bias.

**FIGURE 3:**
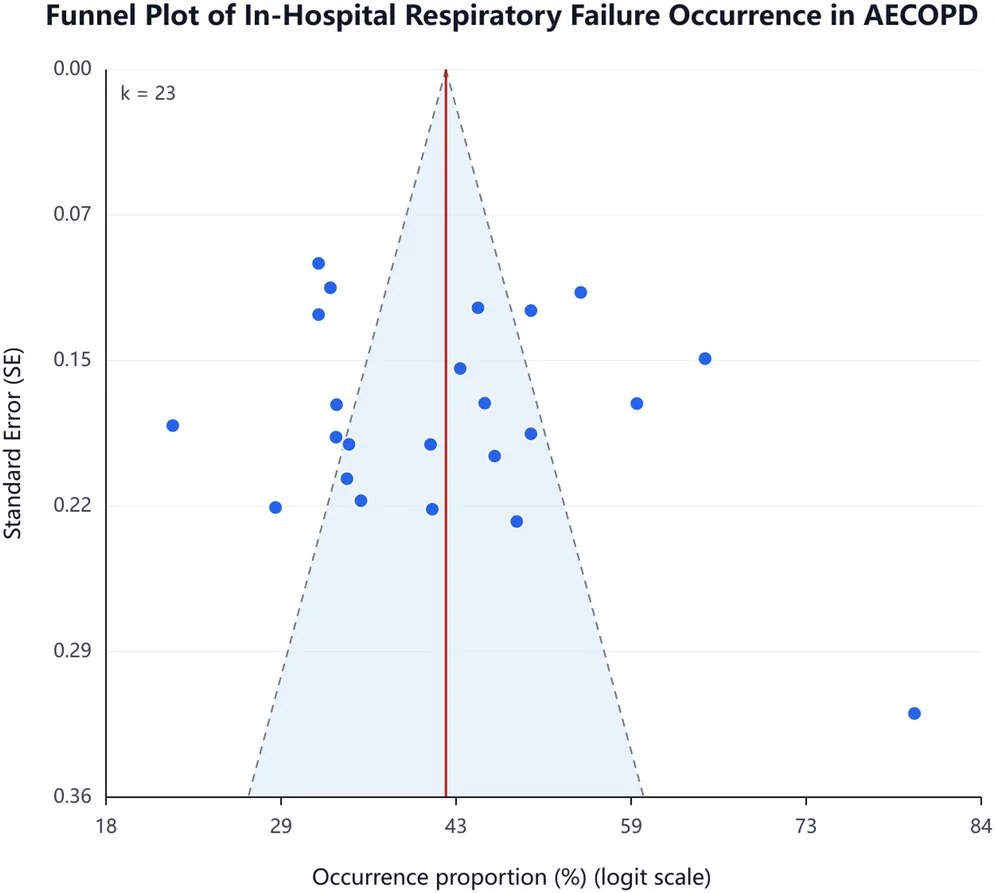
Funnel plot for assessment of publication bias in the meta‐analysis of the occurrence proportion of respiratory failure. Each point represents an individual study, plotted with its effect estimate on the horizontal axis and its standard error on the vertical axis. The red vertical line denotes the pooled estimate on the logit scale. The dashed lines and shaded area represent the pseudo‐95% confidence limits around the pooled estimate, within which studies would be expected to fall in the absence of bias. The distribution of points provides a visual assessment of potential publication bias or small‐study effects in the included studies.

**FIGURE 4:**
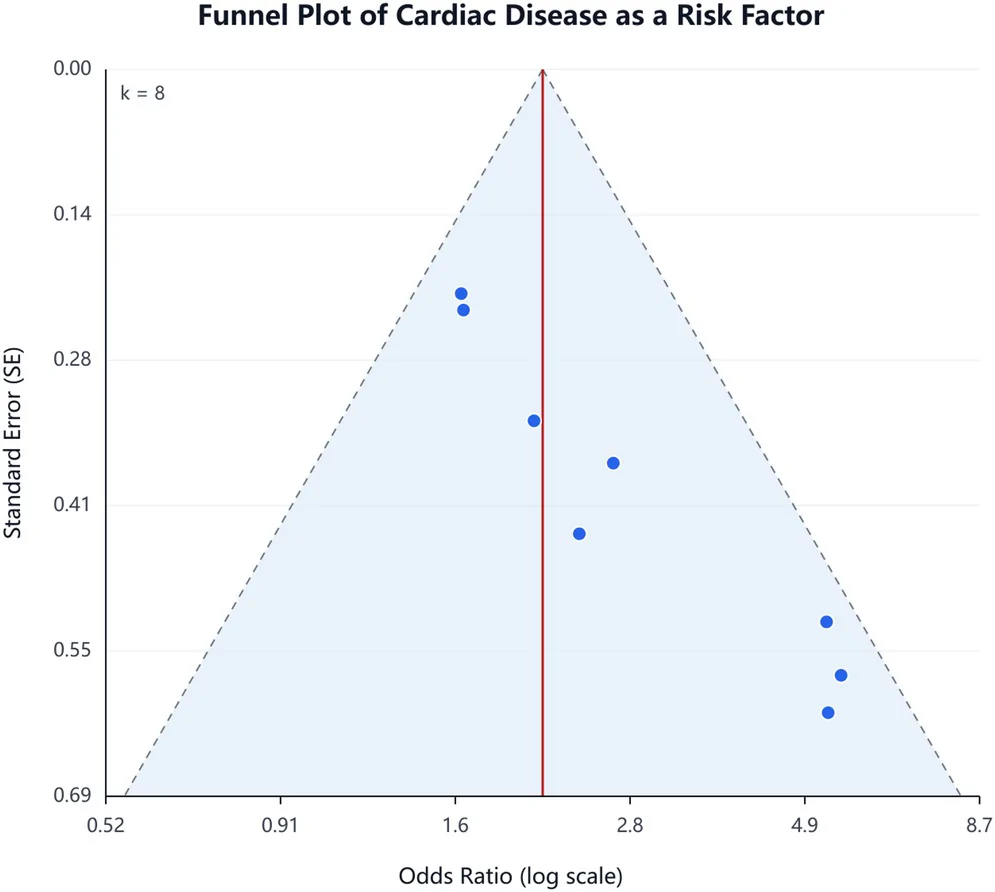
Funnel plot for assessment of publication bias in the meta‐analysis of comorbid cardiac disease as a factor associated with respiratory failure. Each point represents an individual study, plotted with its log‐transformed odds ratio on the horizontal axis and the standard error of the log‐odds ratio on the vertical axis. The red vertical line denotes the fixed‐effect pooled estimate. The dashed lines and shaded area represent the pseudo‐95% confidence limits around the pooled estimate. The plot allows visual assessment of whether smaller studies yield systematically different estimates from larger ones, which may indicate publication bias, though formal tests should be interpreted with caution due to the limited number of studies.

## 4 Discussion

This study systematically integrated the epidemiological and clinical data of 23 observational studies conducted in Chinese populations, involving a total of 4212 hospitalized patients with AECOPD, and the meta‐analysis showed that the occurrence proportion of respiratory failure during hospitalization in Chinese patients with AECOPD was 41%, a relatively high level, suggesting that this complication is highly common in clinical practice. Subgroup analysis showed that, when stratified by study design, the pooled occurrence proportion was 40% in case–control studies and 45% in cohort studies, with no statistically significant difference between subgroups, suggesting that different study design types did not significantly affect the pooled occurrence proportion estimates. When stratified by geographical region, the pooled occurrence proportion of respiratory failure in patients with AECOPD was 48% in the southern region, significantly higher than that in the northern (37%) and central (35%) regions, and the between‐subgroup difference was statistically significant. This regional difference was derived only from study‐level subgroup comparisons and may simultaneously be influenced by differences in single‐center sources, study years, patient composition, case sampling methods, and diagnostic and treatment practices. Differences in hot and humid climates or pathogen spectra may serve as hypotheses requiring further verification, but the current aggregated data cannot establish a causal relationship between these factors and the occurrence proportion of respiratory failure, and study‐level comparisons are subject to ecological fallacy and residual confounding risks. Subgroup analysis stratified by the diagnostic criteria for respiratory failure showed no statistically significant difference in occurrence proportion among the three groups, suggesting that differences in diagnostic criteria for respiratory failure were not a major source of heterogeneity in the occurrence proportion.

At the level of factors associated with respiratory failure, by restricting the analysis to multivariable‐adjusted effect estimates and performing subgroup‐stratified pooling according to indicator definitions and cutoff values, a total of nine statistically significant factors associated with respiratory failure were identified, including comorbid cardiac disease, advanced age, elevated NLR, elevated PCT, elevated D‐dimer, annual exacerbation frequency ≥ 2, higher APACHE II score, elevated CRP, and decreased FEV_1_%pred. It should be noted that ALB did not reach statistical significance in this study; sensitivity analysis showed that the result became statistically significant after using a fixed‐effects model, suggesting that the result under the random‐effects model was unstable, which may be related to differences in the timing of assessments across studies and substantial population heterogeneity. NE was not statistically significant under either model. Therefore, ALB and NE were not included among the nine factors that were statistically significant in the primary analysis.

The study by Chen Huaping et al. [45] showed that respiratory failure was the predominant complication of COPD, accounting for 20.95% of the total study population, which is consistent with the relatively high occurrence proportion of respiratory failure observed in the present study. The publication bias analysis for the occurrence proportion indicated high heterogeneity among the included studies. Subgroup analysis suggested that differences in the geographical distribution of study populations, inclusion and exclusion criteria, and detection methods might all be important sources of heterogeneity. Nevertheless, the pooled occurrence proportion of 41% clearly indicates that respiratory failure is an extremely common serious complication during hospitalization for AECOPD, and clinicians should screen for respiratory failure risk in all high‐risk AECOPD patients, rather than only in those admitted to the intensive care unit (ICU).

The pooled analysis of factors associated with respiratory failure in this study showed that decreased FEV_1_%pred was a factor associated with respiratory failure in patients with AECOPD. Lung function is a core indicator for evaluating the degree of airflow limitation, disease severity, and respiratory reserve capacity in patients with COPD [46]. A lower FEV_1_%pred indicates more severe baseline lung function impairment, irreversible airway structural remodeling, and a sharp increase in ventilatory load after acute exacerbation that far exceeds the body's compensatory capacity, which can readily induce hypoxemia and carbon dioxide retention, ultimately progressing to respiratory failure [47]. Previous studies have shown that the risk of respiratory failure increases significantly when FEV_1_%pred is < 50% [48]. The study by Cui Ting et al. [49] also demonstrated that severe airflow limitation is a high‐risk factor for respiratory failure in elderly patients with AECOPD in the ICU. This result is consistent with the pathophysiological mechanisms and conclusions reported in multiple previous clinical studies, suggesting that baseline lung function level should be used as an important reference for risk stratification of respiratory failure in patients with AECOPD.

Demographic characteristics and underlying comorbidities are important intrinsic factors that drive further progression to respiratory failure in patients with AECOPD. This study suggested that advanced age and comorbid cardiac disease were factors associated with respiratory failure in patients with AECOPD. With advancing age, elderly patients experience physiological decline in respiratory muscle strength, decreased thoracic compliance, weakened cough reflex and airway clearance capacity, and reduced systemic immune defense function; after infection, inflammation tends to spread rapidly and is difficult to control effectively [50, 51]. A meta‐analysis by Yang et al. [52] suggested that age is a significant factor associated with ventilator‐associated pneumonia in patients with AECOPD. Furthermore, a study on a prediction model for in‐hospital mortality among Chinese inpatients with AECOPD also indicated that age was an independent predictor, with nonsurvivors being significantly older than survivors [53]. In the presence of comorbid cardiac disease, hypoxemia and hypercapnia caused by AECOPD can elevate pulmonary artery pressure and increase right ventricular afterload while cardiac dysfunction in turn leads to pulmonary circulatory congestion and pulmonary edema, disrupting the ventilation/perfusion balance; this forms a vicious cycle of cardiopulmonary mutual injury and significantly increases the risk of respiratory failure [54]. A cluster analysis of 526 patients with AECOPD in western China found that the “cardiovascular comorbidity group” had the highest mortality among four clinical phenotypes, at 15.9% [55]. A retrospective analysis of 355 hospitalized patients with AECOPD by Shafi et al. [56] further found that patients with comorbid ischemic heart disease had a significantly increased risk of mortality, and age > 70 years was an important predictor of in‐hospital death.

Acute exacerbation frequency, as a core clinical feature reflecting the level of disease control, was suggested in this study to be an important factor associated with respiratory failure in patients with AECOPD. The presence of ≥ 2 acute exacerbations per year suggests that chronic airway inflammation remains persistently hyperactive, the disease has been poorly controlled over the long term, and repeated inflammatory stimuli continuously aggravate airway injury and disrupt lung tissue structure, leading to progressive decline in lung function. The systematic review by Chamaon et al. [10] clearly indicated that a history of ≥ 1 previous AECOPD episode increased the risk of acute exacerbation in patients with COPD. The meta‐analysis by Liu Xin et al. [57] also found that the annual number of AECOPD episodes was one of the three most consistently identified predictors in risk prediction models for COPD complicated by respiratory failure. Moreover, frequent acute exacerbations can readily induce chronic respiratory muscle fatigue; the body's tolerance to hypoxemia and inflammatory stress continuously declines; and during subsequent acute exacerbations, patients are more likely to exceed the compensatory threshold and progress to respiratory failure [58].

Systemic inflammation and coagulation dysfunction are highly associated with respiratory failure in patients with AECOPD [59]. The results of this study showed that elevated NLR, CRP, PCT, and D‐dimer levels were all independent factors associated with respiratory failure in patients with AECOPD. AECOPD is essentially a systemic inflammatory disease with airway inflammation at its core; during acute exacerbation, pathogen infection can activate massive neutrophil infiltration, trigger a systemic inflammatory cascade, cause lung tissue injury and airway mucosal edema and obstruction, and further impair ventilation and gas exchange [60]. NLR can comprehensively reflect inflammatory activation and immune imbalance in the body, whereas CRP and PCT can sensitively indicate the severity of infection and systemic inflammation; abnormal elevation of all three markers indicates an uncontrolled inflammatory response and is closely associated with the occurrence of respiratory failure [61]. Marked elevation of D‐dimer is closely related to microthrombosis, pulmonary vascular endothelial injury, and ventilation–perfusion mismatch, and is an independent factor associated with the progression of respiratory failure [62].

In terms of critical illness severity scores, this study suggested that a higher APACHE II score is a factor associated with respiratory failure in patients with AECOPD. The APACHE II score integrates acute physiological parameters, age, and chronic health status and can comprehensively and objectively assess the degree of systemic organ dysfunction and the severity of critical illness. A higher score indicates more severe multisystem physiological imbalance, organ functional reserve approaching decompensation, and significantly increased risks of respiratory failure and poor prognosis [63]. The meta‐analysis by Yang et al. [52] showed that the APACHE II score is an important predictor of ventilator‐associated pneumonia in patients with AECOPD. This score can be used clinically for rapid severity stratification of patients with AECOPD and can provide a quantitative reference for intensive care referral, timing of respiratory support, and adjustment of diagnosis and treatment plans, with good clinical utility.

## 5 Limitations

First, the studies included in this meta‐analysis were mainly retrospective observational studies conducted in China; therefore, selection bias and information bias are unavoidable, and moderate‐to‐high heterogeneity remains for some factors associated with respiratory failure, which is mainly related to differences in the geographical regions of study populations, non‐uniform detection methods, varying cutoff value settings, and different confounding adjustment strategies. Although appropriate models were used for pooled analysis, the influence of heterogeneity could not be completely eliminated. Case–control studies select participants by outcome status and cannot estimate incidence. The pooled proportion is therefore descriptive and should not be interpreted as a true incidence. Subgroup analysis by study design showed no significant difference between cohort and case–control studies. This reduces, but does not eliminate, concerns related to case–control sampling. Second, the pooled analyses of the occurrence proportion and some factors associated with respiratory failure were based on the currently available literature; the overall number of studies and sample size were limited, which precluded more detailed subgroup stratified analyses. Third, this study included only domestic studies and lacked support from international multicenter data, which limits the generalizability of the conclusions. In addition, some potential factors associated with respiratory failure could not be quantitatively pooled because the number of studies meeting the inclusion criteria was insufficient, and their associations with respiratory failure in AECOPD remain to be confirmed by more high‐quality studies in the future. Furthermore, the regional subgroup analysis was based on study‐level rather than patient‐level data; region, study design, study year, patient source, and diagnostic and treatment practices may be mutually confounded. Therefore, the higher pooled occurrence proportion in the southern region cannot be directly attributed to climatic or pathogen differences, and caution is warranted regarding ecological fallacy. Model dependence analysis only reflects the dependence of results on the choice of effect model and cannot replace study‐by‐study influence analysis.

## 6 Conclusion

In summary, this study showed that the occurrence proportion of respiratory failure during hospitalization in Chinese patients with AECOPD was 41%, and advanced age, comorbid cardiac disease, annual exacerbation frequency ≥ 2, higher APACHE II score, elevated NLR, CRP, PCT, and D‐dimer levels, and decreased FEV_1_%pred were associated with respiratory failure in patients with AECOPD. Because all included studies were observational and included case–control studies, causality cannot be inferred, and the 41% estimate should not be interpreted as population incidence. Based on these associated factors, clinicians can establish a risk assessment system for respiratory failure in patients with AECOPD, implement early screening, dynamic monitoring of inflammatory markers, standardized management of underlying diseases, and stable‐phase chronic disease management for high‐risk populations, thereby reducing the risk of respiratory failure and improving the overall clinical prognosis and long‐term quality of life of patients with AECOPD.

## Author Contributions

Dandan Liu: study design, data analysis, manuscript writing. Minzuo Chen: study design, data analysis, manuscript writing. Kaosi Wen: data collection, manuscript review. Jingmei Pan: data collection, manuscript review. Xiangyang Duan: data collection, manuscript review. Tao Yang: study supervision, manuscript review. Shengru Tao: study supervision, manuscript review, corresponding author. All authors approved the final version of the manuscript and agree to be accountable for all aspects of this work.

## Funding

The authors report no specific funding for this study.

## Ethics Statement

This study is a systematic review and meta‐analysis of published literature and does not involve direct patient contact or primary data collection. Therefore, institutional ethics approval was not required. This study was conducted in accordance with the ethical standards laid down in the Declaration of Helsinki.

## Conflicts of Interest

The authors declare no conflicts of interest.

## Supporting information


**Table S1:** Basic characteristics and quality assessment of the 23 included studies.
**Table S2:** Model‐dependence analysis of pooled factors associated with respiratory failure in patients with AECOPD.
**Figure S1:** Forest plot of the occurrence proportion of respiratory failure stratified by study design (random‐effects model).
**Figure S2:** Forest plot of the occurrence proportion of respiratory failure stratified by geographical region (random‐effects model).
**Figure S3:** Forest plot of the occurrence proportion of respiratory failure stratified by respiratory failure type (random‐effects model).
**Figure S4:** Forest plot of comorbid cardiac disease as a factor associated with respiratory failure in AECOPD patients (fixed‐effect model).
**Figure S5:** Forest plot of decreased FEV_1_%pred as a factor associated with respiratory failure in AECOPD patients (random‐effects model).
**Figure S6:** Forest plot of advanced age as a factor associated with respiratory failure in AECOPD patients (fixed‐effect model).
**Figure S7:** Forest plot of elevated NLR as a factor associated with respiratory failure in AECOPD patients (fixed‐effect model).
**Figure S8:** Forest plot of elevated PCT as a factor associated with respiratory failure in AECOPD patients (random‐effects model).
**Figure S9:** Forest plot of elevated D‐dimer as a factor associated with respiratory failure in AECOPD patients (random‐effects model).
**Figure S10:** Forest plot of annual exacerbation frequency ≥ 2 as a factor associated with respiratory failure in AECOPD patients (fixed‐effect model).
**Figure S11:** Forest plot of higher APACHE II score as a factor associated with respiratory failure in AECOPD patients (fixed‐effect model).
**Figure S12:** Forest plot of elevated CRP as a factor associated with respiratory failure in AECOPD patients (random‐effects model).
**Figure S13:** Forest plot of ALB as a factor associated with respiratory failure in AECOPD patients (random‐effects model).
**Figure S14:** Forest plot of NE as a factor associated with respiratory failure in AECOPD patients (random‐effects model).

## Data Availability

All data generated or analyzed during this study are included in this published article and its Supporting Information files.
