# Supplementary material for: Occurrence Proportion and Associated Factors of Respiratory Failure During Hospitalization in Chinese Patients With AECOPD: A Systematic Review and Meta‐Analysis
Source: Clin Respir J. 2026 Oct 22;20(10):e70237. doi: 10.1111/crj.70237 (PMC13691177; doi:10.1111/crj.70237)
Supplement: Supplementary file 1 [file CRJ-20-e70237-s001.docx]

**Supplementary Materials**

This file contains 2 supplementary tables and 14 supplementary figures. The tables present detailed characteristics of included studies and model-dependence analysis. The figures present detailed forest plots for subgroup analyses and individual factor meta-analyses.

**List of Supplementary Tables:**

Supplementary Table 1. Basic characteristics and quality assessment of the 23 included studies.

Supplementary Table 2. Model-dependence analysis of pooled factors associated with respiratory failure in patients with AECOPD.

**List of Supplementary Figures:**

Figure S1. Forest plot of the occurrence proportion of respiratory failure stratified by study design (random-effects model).

Figure S2. Forest plot of the occurrence proportion of respiratory failure stratified by geographical region (random-effects model).

Figure S3. Forest plot of the occurrence proportion of respiratory failure stratified by respiratory failure type (random-effects model).

Figure S4. Forest plot of comorbid cardiac disease as a factor associated with respiratory failure in AECOPD patients (fixed-effect model).

Figure S5. Forest plot of decreased FEV₁%pred as a factor associated with respiratory failure in AECOPD patients (random-effects model).

Figure S6. Forest plot of advanced age as a factor associated with respiratory failure in AECOPD patients (fixed-effect model).

Figure S7. Forest plot of elevated NLR as a factor associated with respiratory failure in AECOPD patients (fixed-effect model).

Figure S8. Forest plot of elevated PCT as a factor associated with respiratory failure in AECOPD patients (random-effects model).

Figure S9. Forest plot of elevated D-dimer as a factor associated with respiratory failure in AECOPD patients (random-effects model).

Figure S10. Forest plot of annual exacerbation frequency ≥2 as a factor associated with respiratory failure in AECOPD patients (fixed-effect model).

Figure S11. Forest plot of higher APACHE II score as a factor associated with respiratory failure in AECOPD patients (fixed-effect model).

Figure S12. Forest plot of elevated CRP as a factor associated with respiratory failure in AECOPD patients (random-effects model).

Figure S13. Forest plot of ALB as a factor associated with respiratory failure in AECOPD patients (random-effects model).

Figure S14. Forest plot of NE as a factor associated with respiratory failure in AECOPD patients (random-effects model).

**Supplementary Table 1. Basic characteristics and quality assessment of the 23 included studies.** NOS, Newcastle-Ottawa Scale; a, comorbid cardiac disease; b, D-dimer ; c, Annual exacerbation frequency ≥2; d, neutrophil percentage (NE); e, forced expiratory volume in one second percent predicted (FEV₁%pred); f, age; g, APACHE II score; h, procalcitonin (PCT); i, serum albumin (ALB); j, C-reactive protein (CRP); k, neutrophil-to-lymphocyte ratio (NLR). All studies were conducted in China.

| **First author** | **Year** | **Study type** | **Study period** | **Types of respiratory failure** | **Sample size (cases/controls)** | **Gender (M/F)** | **Risk factors** | **NOS score** |
| --- | --- | --- | --- | --- | --- | --- | --- | --- |
| Li Jingjing ^[22]^ | 2025 | Cohort | 2022-2024 | Mixed respiratory failure | 489(155/334) | 333/156 | a | 6 |
| Li Wendong ^[23]^ | 2025 | Cohort | 2023-2024 | Mixed respiratory failure | 323(176/147) | 259/64 | b | 6 |
| Zhou Xiuye ^[27]^ | 2025 | Case-control | 2019-2024 | Type II respiratory failure | 102(29/73) | 62/40 | c | 6 |
| Yu Ying ^[28]^ | 2025 | Case-control | 2019-2022 | Type II respiratory failure | 160(53/107) | 91/69 | de | 7 |
| Wang Miaomiao ^[24]^ | 2025 | Cohort | 2023-2023 | Type II respiratory failure | 380(124/256) | 235/145 | befgh | 8 |
| Fan Yuanyuan ^[29]^ | 2025 | Case-control | 2022-2024 | Mixed respiratory failure | 186(40/146) | 109/77 | gij | 7 |
| Zeng Lijing ^[25]^ | 2024 | Cohort | 2016-2021 | Type II respiratory failure | 210(137/73) | 132/78 | a | 6 |
| Xia Fei ^[30]^ | 2024 | Case-control | 2020-2021 | Mixed respiratory failure | 274(137/137) | 201/73 | ac | 7 |
| Li Ya ^[31]^ | 2024 | Case-control | 2022-2023 | Type II respiratory failure | 283(128/155) | 193/90 | a | 6 |
| Han Xiangming ^[32]^ | 2024 | Case-control | 2021-2022 | Mixed respiratory failure | 126(43/83) | 60/66 | ghk | 7 |
| Jiao Jingwen ^[33]^ | 2023 | Case-control | 2019-2022 | Type II respiratory failure | 306(97/209) | 250/56 | e | 7 |
| Chen Yan ^[34]^ | 2022 | Case-control | 2019-2021 | Type I respiratory failure | 181(79/102) | 106/75 | fj | 6 |
| Zhang Hui ^[35]^ | 2022 | Case-control | 2017-2020 | Mixed respiratory failure | 78(38/40) | 63/15 | a | 7 |
| Dong Tian ^[36]^ | 2022 | Case-control | 2020-2021 | Type II respiratory failure | 106(36/70) | 86/20 | fhk | 6 |
| He Jiaxiu ^[37]^ | 2022 | Case-control | 2018-2021 | Mixed respiratory failure | 148(88/60) | 80/68 | e | 6 |
| Yin Xiaobin ^[38]^ | 2022 | Case-control | 2018-2020 | Type II respiratory failure | 94(33/61) | 56/38 | d | 7 |
| Xie Shangyu ^[39]^ | 2021 | Case-control | 2018-2020 | Mixed respiratory failure | 107(50/57) | 87/20 | d | 6 |
| Liu Jincai ^[40]^ | 2021 | Case-control | 2019-2020 | Mixed respiratory failure | 120(60/60) | 74/46 | ai | 5 |
| Chen Xiaoliang ^[41]^ | 2021 | Case-control | 2018-2019 | Mixed respiratory failure | 133(44/89) | 120/13 | ac | 6 |
| Ling Ling ^[42]^ | 2020 | Case-control | 2015-2018 | Mixed respiratory failure | 85(35/50) | 51/34 | f | 7 |
| Cheng Lei ^[43]^ | 2020 | Case-control | 2015-2019 | Mixed respiratory failure | 144(66/78) | 88/56 | b | 6 |
| Zhang Lihong ^[26]^ | 2020 | Cohort | 2019-2019 | Mixed respiratory failure | 117(48/69) | 81/36 | k | 5 |
| Zhang Heng ^[44]^ | 2018 | Case-control | 2013-2017 | Type I respiratory failure | 60(30/30) | 47/13 | ai | 6 |

**Supplementary Table 2. Model-dependence analysis of factors associated with respiratory failure in patients with AECOPD.** This table compares the pooled effect sizes and statistical significance of each risk factor under fixed-effects versus random-effects models. ALB, serum albumin; CI, confidence interval; NE, neutrophil percentage; OR, odds ratio.

| **Risk factor** | **Heterogeneity test** | | **Effect model** | **Pooled effect size** | | | |
| --- | --- | --- | --- | --- | --- | --- | --- |
|  | **P-value** | **I^2^%** |  | **OR** | **95%CI** | | **P-value** |
| Combined cardiac disease | 0.12 | 38.61 | Random | 2.38 | 1.72 | 3.27 | 0.00 |
| FEV₁%pred | 0.02 | 69.80 | Fixed | 0.92 | 0.90 | 0.94 | 0.00 |
| Age | 0.41 | 0.00 | Random | 1.22 | 1.13 | 1.33 | 0.00 |
| NLR | 0.32 | 25.76 | Random | 1.90 | 1.46 | 2.47 | 0.00 |
| PCT | 0.02 | 75.59 | Fixed | 1.75 | 1.41 | 2.17 | 0.00 |
| D-dimer | 0.02 | 73.77 | Fixed | 1.42 | 1.22 | 1.64 | 0.00 |
| Annual exacerbation frequency ≥2 | 0.46 | 0.00 | Random | 2.48 | 1.71 | 3.60 | 0.00 |
| APACHE II score | 0.20 | 30.10 | Random | 1.30 | 1.15 | 1.48 | 0.00 |
| CRP | 0.07 | 63.40 | Fixed | 1.08 | 1.04 | 1.11 | 0.00 |
| ALB | 0.00 | 85.00 | Fixed | 0.52 | 0.40 | 0.67 | 0.00 |
| NE | 0.00 | 94.13 | Fixed | 2.07 | 0.90 | 4.79 | 0.09 |

**Figure S1. Forest plot of the occurrence proportion of respiratory failure stratified by study design (random-effects model).**

**Figure S2. Forest plot of the occurrence proportion of respiratory failure stratified by geographical region (random-effects model).**

**Figure S3. Forest plot of the occurrence proportion of respiratory failure stratified by respiratory failure type (random-effects model).**

**Figure S4. Forest plot of comorbid cardiac disease as a factor associated with respiratory failure in AECOPD patients (fixed-effect model).**

**Figure S5. Forest plot of decreased FEV₁%pred as a factor associated with respiratory failure in AECOPD patients (random-effects model).**

**Figure S6. Forest plot of advanced age as a factor associated with respiratory failure in AECOPD patients (fixed-effect model).**

**Figure S7. Forest plot of elevated NLR as a factor associated with respiratory failure in AECOPD patients (fixed-effect model).**

**Figure S8. Forest plot of elevated PCT as a factor associated with respiratory failure in AECOPD patients (random-effects model).**

**Figure S9. Forest plot of elevated D-dimer as a factor associated with respiratory failure in AECOPD patients (random-effects model).**

**Figure S10. Forest plot of annual exacerbation frequency ≥2 as a factor associated with respiratory failure in AECOPD patients (fixed-effect model).**

**Figure S11. Forest plot of higher APACHE II score as a factor associated with respiratory failure in AECOPD patients (fixed-effect model).**

**Figure S12. Forest plot of elevated CRP as a factor associated with respiratory failure in AECOPD patients (random-effects model).**

**Figure S13. Forest plot of ALB as a factor associated with respiratory failure in AECOPD patients (random-effects model).**

**Figure S14. Forest plot of NE as a factor associated with respiratory failure in AECOPD patients (random-effects model).**
